# NCAPD3 exerts tumor-promoting effects in prostatic cancer via dual impact on miR-30a-5p by STAT3-MALAT1 and MYC

**DOI:** 10.1038/s41420-024-01930-7

**Published:** 2024-04-01

**Authors:** Yi Zhang, Yingying Shao, Jia Ren, Yuanyuan Fang, Bolin Yang, Shan Lu, Ping Liu

**Affiliations:** 1 College of Life Sciences, Nanjing Normal University, 210023 Nanjing, Jiangsu P. R. China; 2 Department of Colorectal Surgery, Jiangsu Province Hospital of Chinese Medicine, Affliated Hospital of Nanjing University of Chinese Medicine, 210029 Nanjing, Jiangsu P. R. China

**Keywords:** Oncogenes, Oncogenesis

## Abstract

Non-SMC condensin II complex subunit D3 (NCAPD3) is a subunit of the non-structural maintenance of chromosomes condensin II complex, which involves chromosome condensation and segregation during mitosis. NCAPD3 has recently been demonstrated as a crucial oncogenic factor. However, the underlying mechanism of NCAPD3 in prostate cancer (PCa) remains not completely clear. In this study, we confirmed that lncRNA MALAT1 was induced by NCAPD3-STAT3, and the expression of miR-30a-5p was controlled by NCAPD3 in PCa cells by miRNA-seq. Through quantitative real-time PCR, fluorescence in situ hybridization, western blotting, and immunohistochemistry assay, we demonstrated that miR-30a-5p was lowly expressed in PCa cells and tissues compared to the controls, which was contrary to NCAPD3 expression and markedly downregulated by NCAPD3. Then, MALAT1 was analyzed for the complementary sequence in the potential interaction with miR-30a-5p by using the predicted target module of public databases. Dual-luciferase reporter assay and RNA immunoprecipitation were carried out to verify that MALAT1 functioned as a sponge for miR-30a-5p to reduce miR-30a-5p expression. Meanwhile, MYC acted as a transcriptional repressor to directly bind the promoter of the miR-30a-5p located gene and repress the miR-30a-5p expression. Furthermore, the upregulation of NCAPD3 on cell viability and migration was significantly attenuated in PC-3 cells when miR-30a-5p was overexpressed. NCAPD3 overexpression also accelerated tumor growth in the xenograft mouse model and repressed miR-30-5p. In summary, this work elucidates NCAPD3 inhibits miR-30a-5p through two pathways: increasing STAT3-MALAT1 to sponge miR-30a-5p and increasing MYC to directly inhibit miR-30a-5p transcription, which could serve as potential therapeutic targets for prostate cancer.

## Introduction

Prostate cancer (PCa) is one of the most common malignancies around the world, and among male cancers, its incidence ranked first in the United States and sixth in China [1]. Although a consistent increase of 3.8% occurred during each successive 3-year period in China (2010–2015), there was a huge gap between China (66.4%, 2010–2015) and the United States (97%, 2012–2018) in the 5-year survival rate of prostate cancer [2, 3]. The pathogenesis in PCa encompasses complex factors, which pose significant challenges in developing efficacious treatment strategies. Consequently, the identification of carcinogenetic mechanisms and the establishment of effective therapeutic targets assume paramount importance.

Chromatin structure and topology play crucial roles in the regulation of gene expression, and one feature of the cells with oncogenic potential is the highly dynamic states in chromatin [4]. Both condensin I and condensin II are multi-subunit protein complexes and play major roles in chromosome organization and mitotic chromosome segregation [5], and several subunits of condensins have also been identified as drivers of cancer proliferation [6–8]. Non-SMC condensin II complex subunit D3 (NCAPD3) has initially been known to be only responsible for chromosome condensation and segregation during meiosis and mitosis [9]. In the recent decade, accumulating evidence unveils that NCAPD3 prevents mitochondrial damage and dysfunction [10], down-regulates the transcription of genes encoding amino acid transporters [11], and promotes carcinogenesis [12]. NCAPD3 has been identified as a new biomarker for prostate cancer [13], and we have endorsed this claim based on our previous report that NCAPD3 acted as an androgen/androgen receptor (AR) axis-targeted gene to upregulated lncRNA metastasis-associated lung adenocarcinoma transcript 1 (MALAT1) for accelerating prostate cancer progression through STAT3 [14, 15].

A growing number of studies have proven that lncRNAs can scavenge miRNAs as competing endogenous RNAs or interact with translational machinery by targeting mRNAs, ultimately attenuating miRNAs’ regulatory effect on target mRNAs [16]. MicroRNAs (miRNAs), belonging to a class of non-coding RNAs with approximately 20 nucleotides in length, are usually located in the 3’ untranslated region (3’-UTR) of single-stranded nucleic acids to regulate the expression of genes by binding complementary sequences of specific mRNAs [17]. It has been reported that miR-342-3p was down-regulated to exert oncogenic roles in gallbladder cancer by H19, one of the earliest known lncRNAs [18], and miR-17-5p was also sequestered by H19, resulting in suppressing pro-proliferative mRNAs during myoblast differentiation [19]. Meanwhile, numerous studies have validated that miRNA expression can be regulated by other factors, especially several transcriptional factors (TFs). E2F1 directly bound to miR-223 promoter and inhibited its transcription in AML blast cells [20], Sp1 interacted with miR-29b promoter and thereby repressed miR-29b expression in leukemia cells [21], and miR-22 was repressed at the transcriptional level by Jun in colorectal cancer (CRC) [22]. However, it remained unclear whether there were miRNAs in the downstream regulation of tumor-promoting signaling of NCAPD3-mediated MALAT1 and TFs in PCa.

Here, we identified that miR-30a-5p was downregulated by NCAPD3 through two different pathways, one of which resulted from STAT3 upregulated MALAT1 as a ceRNA for sponging miR-30a-5p, the other was owing to the direct binding of MYC to the promoter of lncRNA LINC00472, the host gene for miR-30a-5p. These effects together significantly weakened the anti-oncogenic role of miR-30a-5p during carcinoma progression.

## Results

### Anomalous expression of NCAPD3 facilitated the upregulation of MALAT1 through the modulation of STAT3 in prostate cancer

**Fig. 1:**
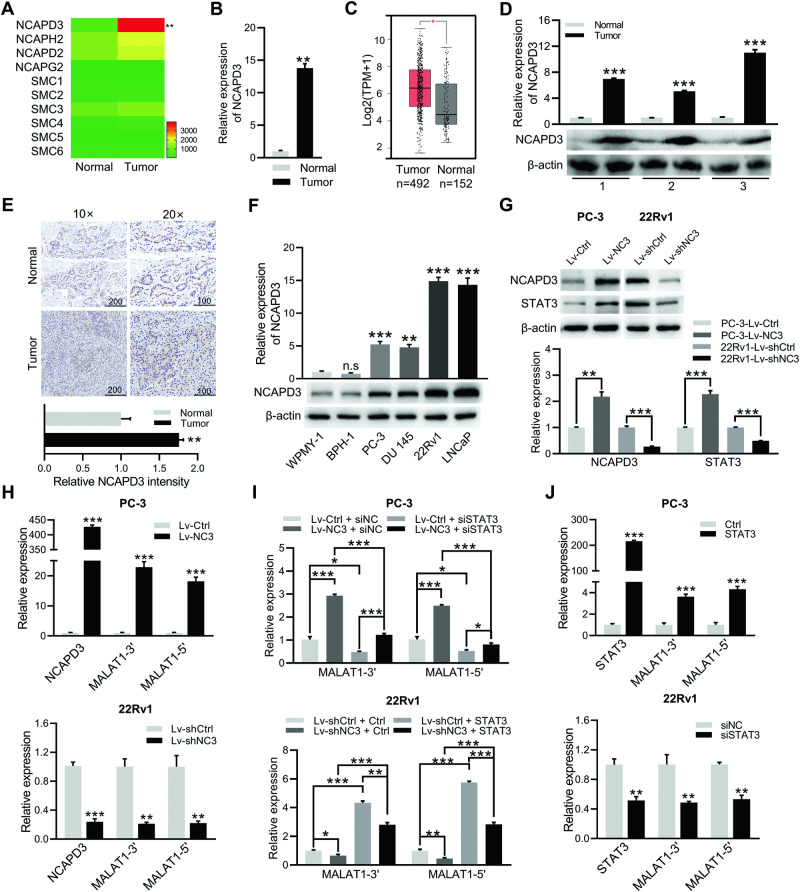
NCAPD3 aberrantly increased in PCa and upregulated MALAT1 expression through STAT3. **A** Ten gene expression profiles of condensin complexes in three pairs of prostate cancer tissues and matched adjacent normal tissues were based on RNA-seq results. **B** qRT-PCR analysis of NCAPD3 mRNA levels in 20 pairs of PCa tissues and adjacent normal tissues. **C** The mRNA levels of NCAPD3 in PCa tissues and normal tissues were analyzed using GEPIA. **D**, **E** The protein levels of NCAPD3 in PCa tissues and adjacent normal tissues were detected by Western blot assay (**D**) and IHC staining (**E**). **F** The protein levels of NCAPD3 in prostate cancer cell lines (PC-3, DU145, 22Rv1, and LNCaP) and non-tumor prostate cell lines (WPMY1 and BPH1) were detected by Western blotting. **G** The protein expression of NCAPD3 and STAT3 was determined by western blot assay in the stable NCAPD3-overexpressing PC-3 cell line (PC-3-Lv-NCAPD3, abbreviated as PC-3-Lv-NC3) or its negative control cell line (PC-3-Lv-Control, abbreviated as PC-3-Lv-Ctrl), and the stable NCAPD3 knockdown 22Rv1 cells (22Rv1-Lv-shNCAPD3, abbreviated as 22Rv1-Lv-shNC3) or its negative control cell line (22Rv1-Lv-shControl, abbreviated as 22Rv1-Lv-shCtrl). **H** qRT-PCR analysis of MALAT1 expression in PC-3-Lv-NCAPD3/Control and 22Rv1-Lv-shNCAPD3/Control. **I** qRT-PCR analysis of MALAT1 expression in transiently transfected cells with STAT3 plasmid (STAT3) or empty vector (Ctrl) in PC-3 or STAT3 siRNA (siSTAT3) or negative control siRNA (siNC) in 22Rv1. **J** qRT-PCR analysis of MALAT1 expression in PC-3-Lv-NCAPD3/Control transiently transfected STAT3 siRNA (siSTAT3) or negative control siRNA (siNC), and in 22Rv1-Lv-shNCAPD3/Control transiently transfected STAT3 plasmid (STAT3) or empty vector (Ctrl). The Western blotting bands were semi-quantitatively analyzed using ImageJ and normalized to β-actin density. Scale bars: 200 and 100 μm, respectively. Normal represents adjacent normal tissues, and Tumor represents prostate cancer patient tissues. Values are means ± SE from *n* = 3 independent repetitions, *p ≤ 0.05, **p ≤ 0.01, and ***p ≤ 0.001 based on two-tailed Student’s *t*-test.

We have previously found that NCAPD3 was highly expressed in PCa and CRC to promote tumor progression through STAT3 upregulation of MALAT1 [15]. Herein, we confirmed the transcription levels of non-SMC subunit NCAPD3, NCAPH2, and NCAPD2 exhibited an increase in tumor tissues compared to adjacent nontumor tissues by RNA-seq analysis, among which only NCAPD3 showed a statistically significant change (Fig. 1A). qRT-PCR analysis of twenty paired PCa tissues displayed that NCAPD3 was similarly upregulated in tumor tissues (Fig. 1B). Gene expression analysis of NCAPD3 in bulk tumor samples from TCGA database using GEPIA web tool (http://gepia.cancer-pku.cn/) were consistent with RNA-seq and qRT-PCR analyses (Fig. 1C). Both of Western blotting and IHC showed the level of NCAPD3 protein was higher in PCa tissues than normal tissues (Fig. 1D, E). Compared to human prostate stromal WPMY1 cells and benign prostatic hyperplasia BPH1 cells, all tumor cell lines (PC-3, DU 145, 22Rv1 and LNCaP) significantly increased NCAPD3 expression (Fig. 1F). To investigate the effect of NCAPD3 on prostate cancer development, we established stable NCAPD3-overexpression PC-3 cells (PC-3-Lv-NC3) and stable NCAPD3-knockdown 22Rv1 cells (22Rv1-Lv-shNC3). We also confirmed that NCAPD3 positively regulated STAT3 and MALAT1 expression in these cells (Fig. 1G, H; Supplementary Fig. 1A). Transient knockdown of STAT3 caused a significant fall in MALAT1 level in PC-3-Lv-NC3 (Fig. 1I, upper; Supplementary Fig. 1B). Conversely, MALAT1 markedly rose in 22Rv1-Lv-shNC3 with transient overexpressed STAT3 (Fig. 1I, lower; Supplementary Fig. 1B). Likewise, MALAT1 was upregulated by STAT3 overexpression in wild-type PC-3 and downregulated by STAT3 knockdown in wild-type 22Rv1 cells (Fig. 1J).

### The modulation of miRNA expression profiles by NCAPD3 in prostate cancer cells was measured by miRNA-seq

**Fig. 2:**
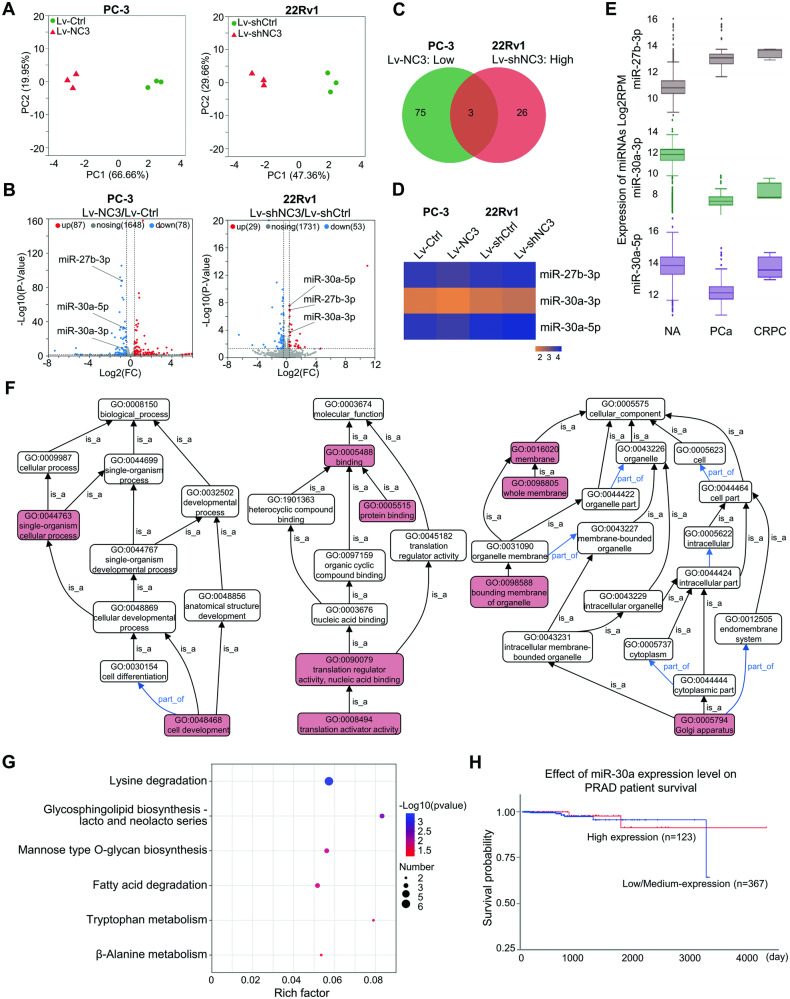
Identification of differentially regulated miRNAs associated with NCAPD3. **A** PCA analysis was conducted based on miRNA-Seq data from the four cell lines (PC-3-Lv-NC3, PC-3-Lv-Ctrl, 22Rv1-Lv-shNC3, and 22Rv1-Lv-shCtrl). **B** Volcano plot of miRNA-seq profile using DEseq2. Differential miRNA expression was shown by red (upregulation) versus blue (downregulation) intensity. **C**, **D** Venn diagram representing the overlap between down-regulated differential miRNAs in PC-3-Lv-NC3/PC-3-Lv-Ctrl and upregulated differential miRNAs in 22Rv1-Lv-shNC3/22Rv1-Lv-shCtrl (**C**). Heat map analysis of the three differential miRNAs from the intersection (**D**). **E** The three differential miRNA expression profiles in different prostate tissues were analyzed on miTED database. **F** The directed acyclic graph (DAG) of GO analysis of miR-30a target genes. Enriched pathways labeled in red reached significance (*p* < 0.05). Arrows indicate the following ontology relationships: black--is_a, blue--part_of. **G** KEGG pathway enrichment analysis was conducted to characterize the miR-30a target genes. **H** Overall survival based on miR-30a expression in TCGA-miRNA prostate cancer cohort was performed by Kaplan–Meier analysis (using UALCAN software).

LncRNAs can act as miRNA sponges and regulate the availability of miRNA for binding mRNA targets [16]. We posited there might be specifical miRNAs involved in the signaling pathway of NCAPD3-STAT3-MALAT1 in prostate cancer. To verify our conjecture, we analyzed the miRNA-seq transcriptome of PC-3-Lv-NC3 and 22Rv1-Lv-shNC3 cells, along with their respective control cells (Supplementary Fig. 2A). Principal component analysis showed that the combined contributions of PC1 and PC2 accounted for over 60% of the total variance (86.61% in PC-3 cells, 77.02% in 22Rv1 cells), indicating a clear distinction between the cell lines (Fig. 2A). We performed differential expression analysis with the R packages of DESeq2 bioconductor to identify the differentially expressed miRNAs between NCAPD3 overexpression or knockdown and control cells. The screening criteria were *p* < 0.05 with FC < 0.77 or FC > 1.30. We detected more than 1000 known miRNAs in each cell line (Fig. 2B). When compared to control cells, respectively, 27 miRNAs significantly increased, and 28 miRNAs decreased in PC-3-Lv-NC3 cells, while 29 miRNAs considerably rose, and 53 miRNAs reduced in 22Rv1-Lv-shNC3 cells. Only three miRNAs were identified in the intersecting set which were not only downregulated in PC-3-Lv-NC3 cells but also upregulated in 22Rv1-Lv-shNC3 cells. They were hsa-miR-27b-3p, hsa-miR-30a-3p, and hsa-miR-30a-5p, and visually represented through a heatmap (Table 1, Fig. 2D). We further found that miR-30a-3p and miR-30a-5p were downregulated in primary prostate cancer and castration-resistant prostate cancer (CRPC) by analyzing the microRNA tissue-expression database miTED (https://dianalab.e-ce.uth.gr/mited/) (Fig. 2E), whereas miR-27b-3p expression was increased in primary prostate cancer and CRPC. Therefore, we focused on miR-30a-3p and miR-30a-5p in the following experiments. As miR-30a-3p and miR-30a-5p were produced from the same precursor miR-30a, we then used three GO-directed acyclic graphs including three categories (biological process, molecular function, and cellular component) to visualize the GO nodes (terms) enriched by miR-30a target genes and their hierarchical relationships. The result showed ten significantly enriched GO terms: cell development (GO:0048468), single-organism cellular process (GO:0044763), translation activator activity (GO:0008494), translation regulator activity, nucleic acid binding (GO:0090079), protein binding (GO:0005515), binding (GO:0005488), bounding membrane of organelle (GO:0098588), membrane (GO:0016020), whole membrane (GO:0098805), Golgi apparatus (GO:0005794) (Fig. 2F; Supplementary Fig. 2B). Subsequently, KEGG pathway enrichment analysis revealed that the significant pathways of miR-30a target genes were associated with Lysine degradation, Glycosphingolipid biosynthesis-lacto and neolacto series, Mannose type O-glycan biosynthesis, Fatty acid degradation, Tryptophan metabolism and beta-Alanine metabolism (*p* < 0.05) (Fig. 2G). Kaplan–Meier survival analysis using the UALCAN database showed that low expression of miR-30a was associated with poor survival probability (Fig. 2H).

**Table 1:** The expression profiles of hsa-miR-27b-3p, hsa-miR-30a-3p, and hsa-miR-30a-5p from miRNA-seq.

miRNA	FPKM (mean value)		log_2_FC	*p*-value
	PC-3-Lv-Control	PC-3-Lv-NCAPD3		
hsa-miR-27b-3p	7045.54	4336.04	−0.85	2.75E−88
hsa-miR-30a-3p	122.05	76.77	−0.82	1.16E−09
hsa-miR-30a-5p	7926.25	5486.23	−0.68	1.07E−33
**miRNA**	**FPKM (mean value)**		**log**_**2**_**FC**	***p*-value**
	**22Rvl-Lv-shControl**	**22Rvl-Lv-shNCAPD3**		
hsa-miR-27b-3p	7551.99	10034.84	0.41	1.15E−07
hsa-miR-30a-3p	169.48	239.19	0.50	2.06E−04
hsa-miR-30a-5p	11756.80	15705.51	0.42	2.75E−08

High-quality total RNA extracted from PC-3-Lv-Control, PC-3-Lv-NCAPD3, 22Rv1-Lv-shControl, and 22Rv1-Lv-shNCAPD3 were submitted to miRNA sequencing at Majorbio (Shanghai, China). The three miRNAs (hsa-miR-27b-3p, hsa-miR-30a-3p, and hsa-miR-30a-5p) were ultimately identified as being significantly downregulated in PC-3-Lv-NC3 cells and significantly upregulated in 22Rv1-Lv-shNC3 cells. Fragments per kilobase million (FPKM) value indicated the relative expression of miRNAs.

Combining our miRNA sequencing with the analysis of publicly available databases, we considered that the NCAPD3–STAT3–MALAT1 pathway could be involved in the inhibition of miR-30a-3p and miR-30a-5p or their precursor miR-30a in PCa, which might relate to cell development, transcription activation, and nucleic acid or protein binding.

### NCAPD3 significantly suppressed the expression of miR-30a-5p in prostate cancer

**Fig. 3:**
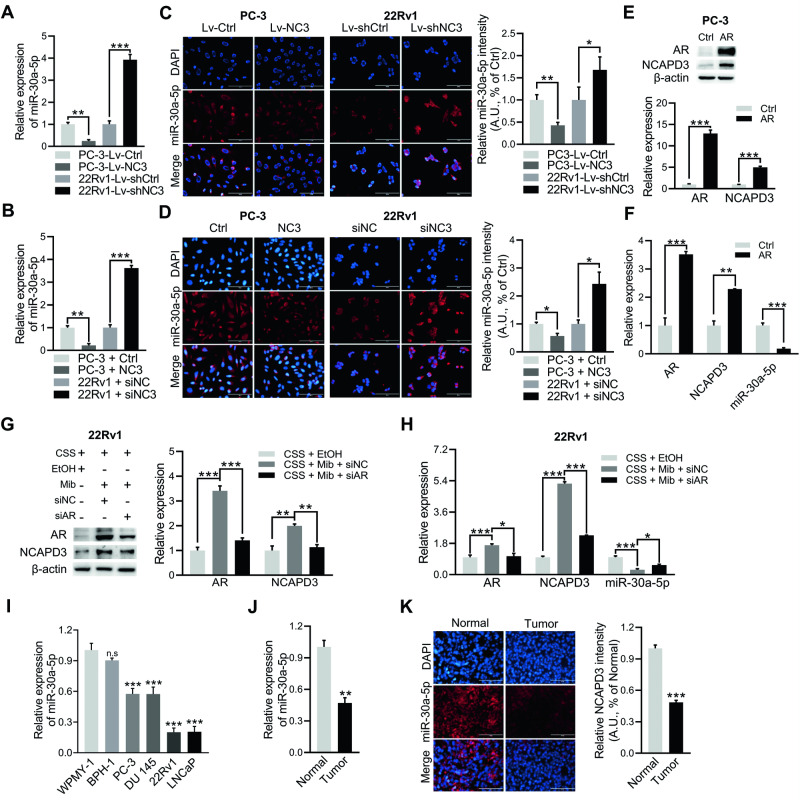
NCAPD3 significantly inhibited miR-30a-5p expression in prostate cancer. **A**, **B** qRT-PCR analysis of miR-30a-5p expression in stable NCAPD3 overexpression or knockdown cells (**A**) and transiently transfected cells with NCAPD3 plasmid or siRNA (**B**). **C**, **D** Detection of miR-30a-5p labeled by Cy3 (red) by RNA FISH in stable NCAPD3 overexpression or knockdown cells (**C**) and transiently transfected cells with NCAPD3 plasmid or siRNA (**D**). **E** The expression efficiency of AR in PC-3 transiently transfected with AR plasmid was demonstrated by Western blot assay. **F** qRT-PCR analysis of AR, NCAPD3, and miR-30a-5p expression in PC-3 transiently transfected with AR plasmid. **G**, **H** 22Rv1 cells were transfected siAR or siNC and incubated with phenol-red-free media containing 10% CSS for 48 h, then treated with or without 10 nM Mib for another 24 h. Protein levels of AR, NCAPD3, and β-actin were checked by Western blotting (**G**). qRT-PCR analysis of miR-30a-5p expression was detected in cells (**H**). **I** qRT-PCR analysis of miR-30a-5p expression in PC-3, DU 145, 22Rv1, LNCaP, WPMY-1 and BPH-1. **J**, **K** Detection of miR-30a-5p expression in PCa tissues and adjacent normal tissues by qRT-PCR (**J**) and FISH (**K**). Scale bars: 100 μm. Normal represents adjacent normal tissues, Tumor represents prostate cancer patient tissues. Values are means ± SE from *n* = 3 independent repetitions, *p ≤ 0.05, **p ≤ 0.01, and ***p ≤ 0.001 based on two-tailed Student’s *t*-test.

To ascertain the regulatory effect of NCAPD3 on miR-30a-5p and miR-30a-3p, we first used qRT-PCR to measure the expression levels of these miRNAs in stable NCAPD3 overexpression PC3 cells and stable NCAPD3 knockdown 22Rv1 cells (Fig. 3A; Supplementary Fig. 3A, B), which showed both of miRNAs were elevated in 22Rv1-Lv-shNC3 cells and reduced in PC3-Lv-NC3. Subsequently, we focused on elucidating the molecular mechanism of NCAPD3 signaling in regulating miR-30a-5p since its expression was varied more significantly by NCAPD3. qRT-PCR similarly revealed that NCAPD3 hindered miR-30a-5p expression in cell lines transiently overexpressing or knocking down NCAPD3 (Supplementary Fig. 3C; Fig. 3C). This was confirmed to detect Cy3-labeled miR-30a-5p more intuitively by FISH in PCa cell lines transiently and stably transfected with NCAPD3 (Fig. 3C, D), where the fluorescence intensity of miR-30a-5p exhibited a strong negative correlation with the expression of NCAPD3 in all cells. Our previous data also demonstrated that AR upregulated the expression of NCAPD3 in prostate cancer [14]. Thus, we further sought to investigate whether AR could affect the expression of miR-30a-5p. Contrary to AR upregulation of NCAPD3, AR overexpression dramatically decreased miR-30a-5p expression in PC-3 cells, while AR knockdown reduced NCAPD3 and improved miR-30a-5p in 22Rv1 cells (Fig. 3E–H). Next, we examined miR-30a-5p expression in all six prostate cell lines (nontumor cell lines: WPMY-1 and BPH-1; tumor cell lines: PC-3, DU 145, 22Rv1 and LNCaP) using qRT-PCR analysis. All tumor cells exhibited a significant decrease in miR-30a-5p expression compared to non-tumor cell lines WPMY-1 and BPH-1, especially LNCAP and 22Rv1 (Fig. 3I), which was exactly contrary to NCAPD3 expression in cells (Fig. 1F). Likewise, the level of miR-30a-5p was significantly elevated in adjacent normal tissues compared to tumor tissues (Fig. 3J), and the FISH result showed the same trends (Fig. 3K). Therefore, miR-30a-5p decreased in PCa and was regulated by NCAPD3 and AR.

### NCAPD3 downregulated miR-30a-5p through MALAT1 as a miRNA sponge

**Fig. 4:**
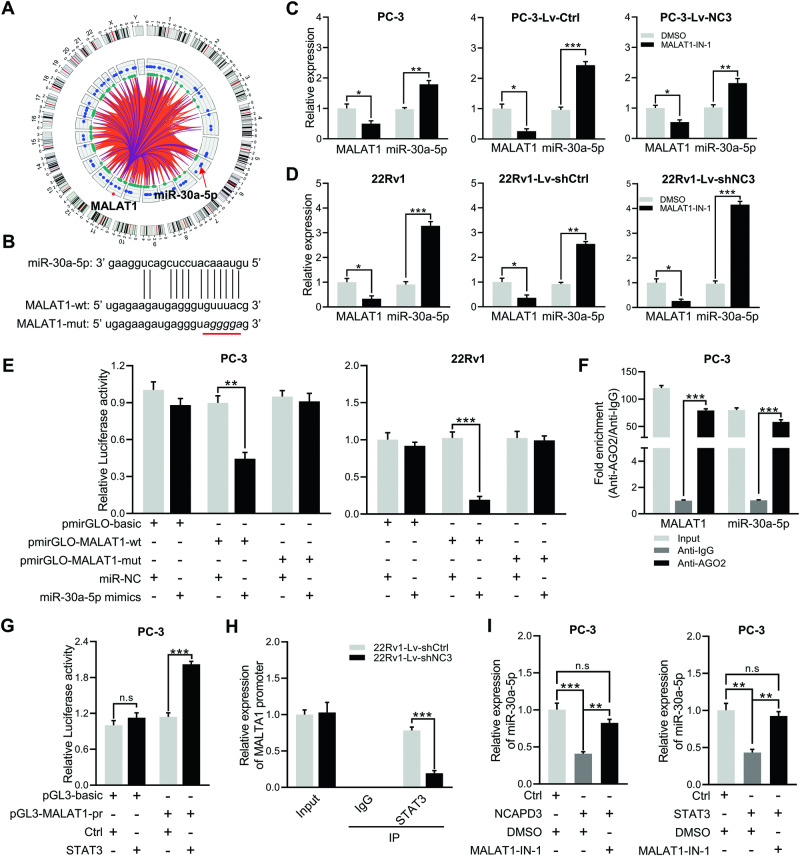
NCAPD3–STAT3–MALAT1 signaling axis significantly decreased miR-30a-5p level. **A** Prediction of the ceRNA networks about MALAT1 in prostate cancer was conducted using LNCAR database. **B** The predicted miR-30a-5p binding sites in MALAT1 (MALAT1-wt) screening from starBase and the designed mutant sequence (MALAT1-mut) were indicated. **C**, **D** qRT-PCR analysis of miR-30a-5p expression in PC-3 and 22Rv1 cells treated with MALAT1-IN-1 (10 µM) or DMSO. **E** The binding between MALAT1 and miR-30a-5p was validated using the dual-luciferase reporter assay in PC-3 and 22Rv1 cells. **F** AGO2-RIP analysis of the binding between MALAT1 or miR-30a-5p and AGO2 in PC-3 cells. **G** The detection of STAT3 binding to the MALAT1 promoter was accomplished by utilizing a dual-luciferase reporter assay in PC-3 cells. **H** PC-3 cells were processed for ChIP experiments with control IgG or antibodies against STAT3, followed by qRT-PCR using primers for the MALAT1 promoter region. **I** qRT-PCR analyzed miR-30a-5p expression in PC-3 cells transiently transfected cells with NCAPD3 (left) or STAT3 (right) plasmid and treated with 10 µM MALAT1-IN-1 or DMSO. Values are means ± SE from *n* = 3 independent repetitions, *p ≤ 0.05, **p ≤ 0.01, ***p ≤ 0.001 and ****p ≤ 0.0001 based on two-tailed Student’s *t*-test.

To verify whether MALAT1 could affect downstream gene expression through sequestering target miRNAs under the regulation of NCAPD3-STAT3 signaling, the ceRNA network diagram was drawn using the LncRNA-miRNA-mRNA module in LNCAR (https://lncar.renlab.org/) as shown in Fig. 4A. The presence of miR-30a-5p in the network graph led us to infer that MALAT1 might directly interact with miR-30a-5p, and the predicted binding sites between MALAT1 and miR-30a-5p were speculated using StarBase (http://starbase.sysu.edu.cn/) (Fig. 4B). qRT-PCR was performed in wide type and genetically engineered PC-3 and 22Rv1 cells with the treatment of a specific MALAT1 inhibitor (MALAT1-IN-1, 10 µM), which showed miR-30a-5p was increased along with decreasing MALAT1 expression (Fig. 4C, D). To detect whether miR-30a-5p could combine with MALAT1, we constructed luciferase reporter plasmids inserted with MALAT1 wild-type and mutant binding sequences (pmirGLO-MALAT1-wt and pmirGLO-MALAT1-mut) to carry out the dual-luciferase reporter assays (Fig. 4E). The relative luciferase activity (RLU) was declined when wild-type MALAT1 plasmid was co-transfected with miR-30a-5p mimics in PC-3 and 22Rv1 for 48 h, whereas it was not changed by co-transfection of mutant MALAT1 and miR-30a-5p mimics. In addition, RNA immunoprecipitation assay (RIP) showed that both MALAT1 and miR‐30a‐5p were immunoprecipitated with AGO2 antibody in PC-3 (Fig. 4F). Moreover, STAT3 was confirmed to regulate luciferase MALAT1 promoter activity in PC-3 cells by dual-luciferase reporter assay and bind with the MALAT1 promoter in stable 22Rv1-knockdown cells by ChIP-PCR using a STAT3 antibody (Fig. 4G, H). Finally, we found that the reduction of miR-30a-5p resulting from NCAPD3 or STAT3 overexpression could be partially rescued in PC-3 treated with MALAT1-IN-1 (Fig. 4I). The aforementioned result demonstrated that NCAPD3 inhibited miR-30a-5p through STAT3-regulated MALAT1 as a miRNA sponge.

### NCAPD3 downregulated miR-30a-5p through MYC as a transcriptional repressor

**Fig. 5:**
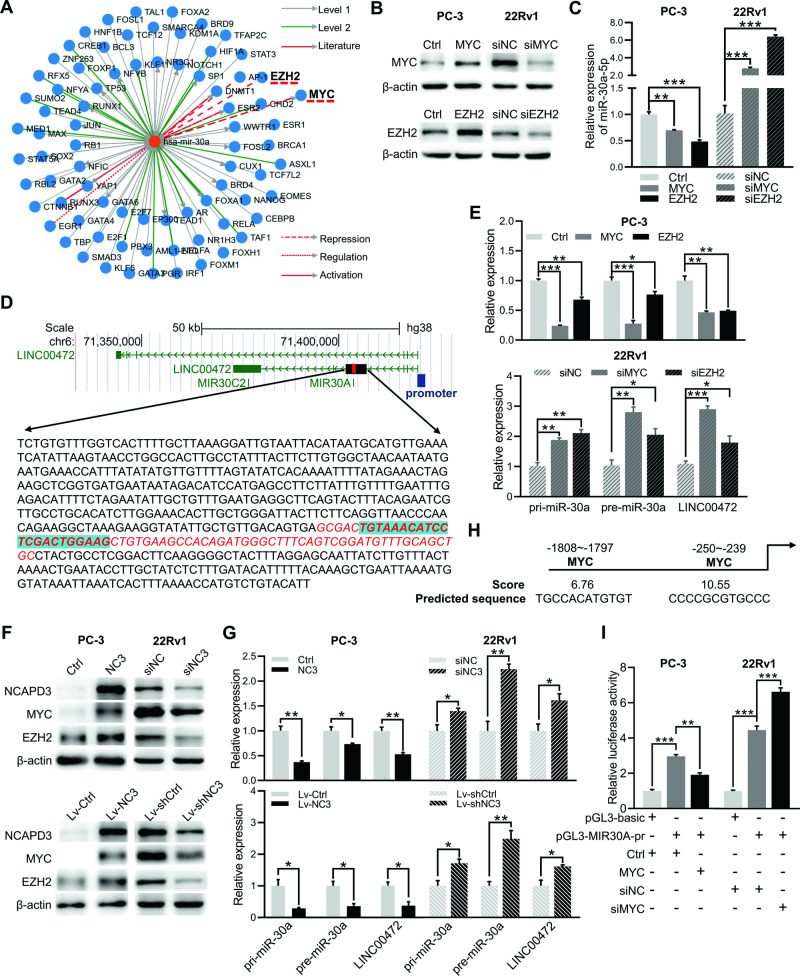
NCAPD3 mediated MYC inhibited transcription of miR-30a by binding its promoter. **A** Transcription factors regulated miR-30a were predicted from TransmiR. “Level 1/2” and “literature” represented the degree of confidence in TF-miRNA regulation. All literature-curated TF-miRNA regulations were annotated as “literature”, and the regulations derived from ChIP-seq were annotated as “level 1/2” (level 2 promoter was supported by high-throughput experimental data). **B**, **C** The expression efficiency of MYC and EZH2 in PC-3 and 22Rv1 transiently transfected with plasmid or siRNA was demonstrated by Western blotting (**B**), and qRT-PCR analyzed miR-30a-5p expression in these cells (**C**). **D** A zoomed-in view of the genome position and sequence of pri-miR-30a located within LINC00472 from the UCSC genome browser. **E** qRT-PCR analysis of miR-30a-5p expression in PC-3 (Upper) and 22Rv1 (Lower) cells transiently transfected with MYC and EZH2 plasmid or siRNA. **F**, **G** Protein expression of MYC and EZH2 were determined by western blot assay in cells transiently transfected with NCAPD3 plasmid or siRNA and in stably transfected NCAPD3 or NCADPD3 knockdown cells (**F**). qRT-PCR analyzed pri-miR-30a, pre-miR-30a, and LINC00472 expression in these cells (**G**). **H** The binding sites of transcription factor MYC in the promoter of LINC00472 were predicted on the JASPAR database. **I** Binding of MYC to LINC00472 promoter detected by dual-luciferase reporter assay in PC-3 cells. Values are means ± SE from *n* = 3 independent repetitions, *p ≤ 0.05, **p ≤ 0.01, and ***p ≤ 0.001 based on two-tailed Student’s *t*-test.

Although miR-30a-3p expression did not have experimentally significant change, it was inhibited by NCAPD3 as similar to miR-30a-5p level in RNA-seq analysis, and both miRNAs were processed from the same precursor miR-30a. Hence, we speculated that NCAPD3 might inhibit miR-30a-5p expression through transcriptional and post-transcriptional regulation, except for increasing MALAT1 as a miRNA sponge. The upstream transcription factors of miR-30a were predicted by using JASPAR, seven of which had been reported, including UNX3, EGR1, AP-1, EZH2, MYC, ESR2, and DNMT1, and the last five among them may behave as transcriptional repressors (Table 2). Meanwhile, miR-30a was predicted again to be suppressed by the transcription factor MYC and EZH2 through the TransmiR v2.0 (http://www.cuilab.cn/transmir) (Fig. 5A). We noted a significant reduction in the expression of miR-30a-5p upon MYC and EZH2 overexpression in PC-3 cells, respectively, while a substantial increase was observed following MYC and EZH2 knockdown in 22Rv1 cells (Fig. 5B, C). Based on the reported literature [23], we identified the sequences of pri-miR-30a and pre-miR-30a were located within the intron of LINC00472 and subsequently designed specific primers of them (Fig. 5D, Supplementary Table 2). qRT-PCR demonstrated that pri-miR-30a, pre-miR-30a, and LINC00472 were decreased when MYC and EZH2 were overexpressed in PC-3 cells, and increased after MYC and EZH2 knockdown in 22Rv1 cells (Fig. 5E). Our recent research reported that NCAPD3 regulated EZH2 expression [15], and MYC expression was also verified to be regulated by NCAPD3 (Fig. 5F; Supplementary Fig. 3D). Importantly, pri-miR-30a, pre-miR-30a and even LINC00472 were all inhibited by NCAPD3 in PCa cells (Fig. 5G). Therefore, we surmised that NCAPD3 inhibited the transcription initiation of LINC00472, or rather, the transcription initiation of miR-30a. Two potential transcription factor MYC binding sites to the miR-30a promoter region (chr6:71420148-71422096) were found using JASPAR, which were located at around 239 and 1797 bp upstream of the transcription start site (Fig. 5H). Then, we constructed a luciferase reporter plasmid containing the miR-30a promoter for co-transfection with MYC plasmid or siRNA. As shown in Fig. 5I, the luciferase activity of miR-30a promoter was significantly suppressed when MYC was overexpressed in PC-3, whereas it was enhanced when MYC was knocked down in 22Rv1. From the obtained information, we concluded that NCAPD3-induced MYC inhibited the expression of miR-30a-5p by controlling transcription initiation.

**Table 2:** Transcription factors regulated miR-30a-5p were identified from JASPAR.

target pre-miRNA name	TF	TF_gene_id	*p*-value	Regulation
hsa-mir-30a	RUNX3	ENSG00000020633	1.05E−05	Activation
	EGR1	ENSG00000120738	2.20E−04	Regulation
	AP-1	ENSG00000177606		Repression
	EZH2	ENSG00000106462		Repression
	MYC	ENSG00000136997	1.45E−04	Repression (feedback)
	ESR2	ENSG00000140009	2.76E−05	Repression (feedback)
	DNMT1	ENSG00000130816		Repression (feedback)

List of transcription factors that were identified by the Jaspar tool analyzing the 2000 bp upstream sequence of hsa-mir-30a.

### NCAPD3 promoted PCa cell proliferation and migration in vitro by enhancing the expression of STAT3-MALAT1 and declining the level of miR-30a-5p

**Fig. 6:**
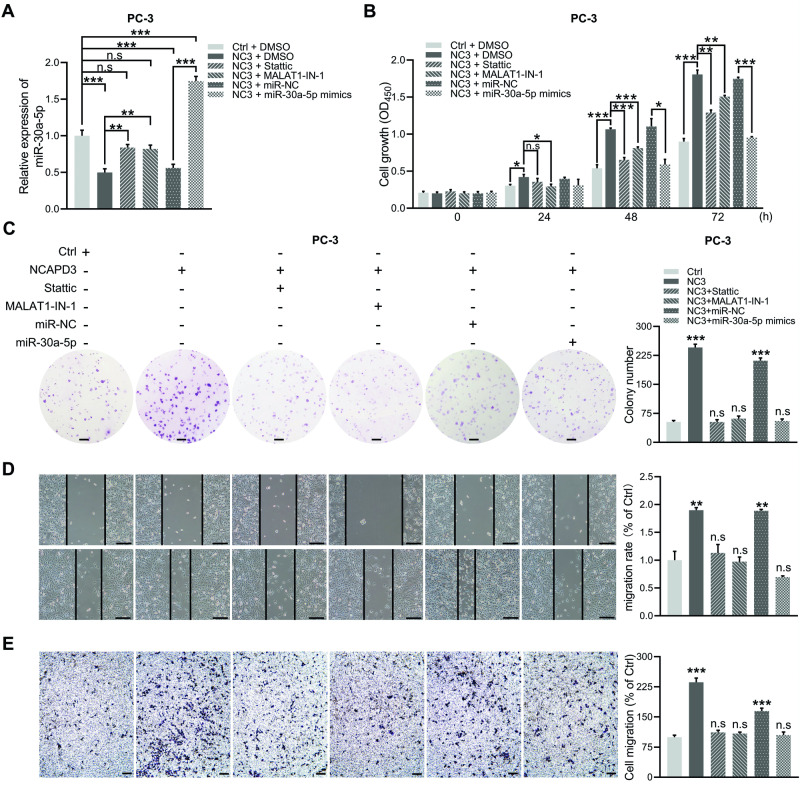
NCAPD3 promoted the proliferation and migration of PCa cells via inhibition of miR-30a-5p. **A** The expression level of miR-30a-5p was detected by qRT-PCR in stable NCAPD3 overexpression PC-3 cells with different transfections and inhibitor treatment as indicated. MALAT1-IN-1: 10 µM; Stattice: 5 µM; miRNA mimic: 50 nM. **B**, **C** Cell viability was measured by CCK-8 assay (**B**) and colony formation analysis (**C**). **D**, **E** Wound-healing assays (**D**) and transwell migration assays (**E**) were conducted to evaluate cell migration ability. Scale bars: 200 μm. Values are means ± SE from *n* = 3 independent repetitions, *p ≤ 0.05, **p ≤ 0.01, and ***p ≤ 0.001 based on two-tailed Student’s *t*-test.

To investigate the impact of STAT3 and MALAT1 on NCAPD3 inhibition of miR-30a-5p and the effect of NCAPD3-STAT1-MALAT1-miR-30a-5p pathway on prostate cancer cell proliferation and migration, stable NCAPD3 overexpression PC-3 cells were individually treated with STAT3 specific inhibitor, Stattic, MALAT1 inhibitor, MALAT1-IN-1, or transfected with miR-30a-5p mimics. qRT-PCR shown that NCAPD3-dowregulated miR-30a-5p expression were obviously rescued by either inhibitor for STAT3 or MALAT1, and to a much higher degree by the transfection of miR-30a-5p mimics compared to negative control miRNA (Fig. 6A). The CCK-8 assays demonstrated that the NCADP3 overexpression enhanceed cell viability was partly decreased by STAT3 inhibitor and MALAT1 inhibitor, respectively, and totally dropped by miR-30a-5p mimics (Fig. 6B). The colony-forming assays showed that NCAPD3 overexpression promoted cell colony-forming, while the enhancement was suppressed with stattic, MALAT1-IN-1 or miR-30a-5p mimics (Fig. 6C). Images of the cratch wound assays showed the wound-healing ability was significantly reduced in NCAPD3 overexpression cells by the treatment of STAT3 inhibitor, MALAT1 inhibitor, or miR-30a-5p mimics (Fig. 6D). In line with the above functional experiments, transwell migration assays also demonstrated that cell migration was significantly decreased by stattic, MALAT1-IN-1 or miR-30a-5p mimics (Fig. 6E).

### miR-30a-5p was downregulated in NCAPD3-induced promoting tumor growth in the subcutaneous xenograft mouse model of prostate cancer

**Fig. 7:**
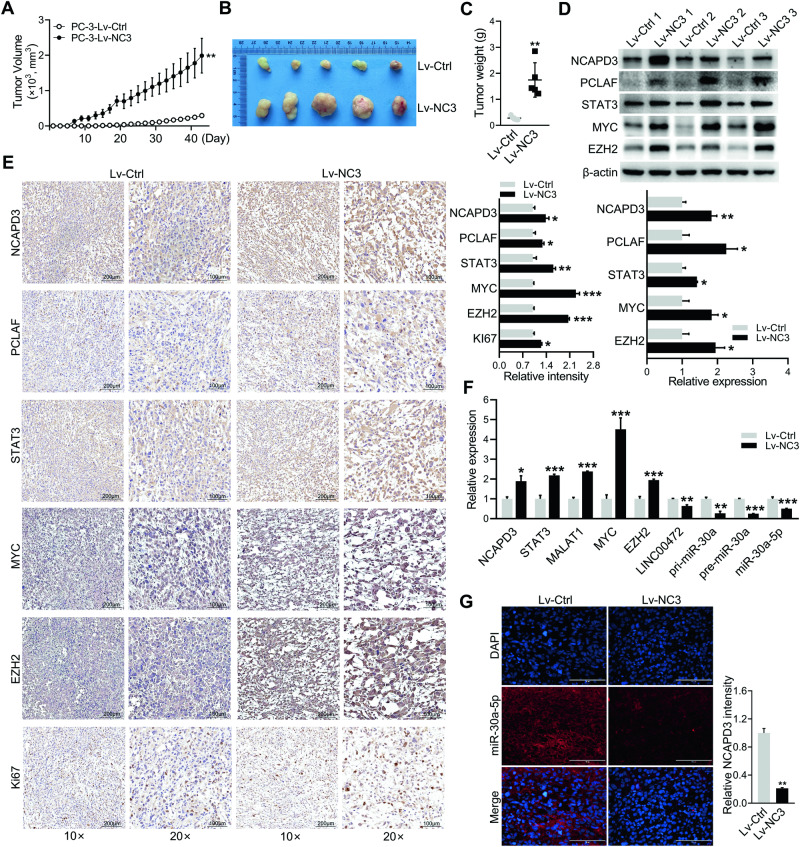
NCAPD3 promoted tumor growth by inhibiting miR-30a-5p expression in the subcutaneous xenograft mouse model. **A** Tumor volume changed in nude mice injected with PC-3 cells with stable NCAPD3 overexpression and empty vector control from day 0 to day 43. **B** Tumor images were dissected from the nude mice after being subcutaneously injected. **C** Tumor weight of each mice group was measured and presented. **D** Protein expression of genes was determined by western blot assay in xenograft tumors. **E** Representative images of IHC staining of NCAPD3, STAT3, MYC, EZH2, PCLAF, and Ki-67 on tumor sections. **F** qRT-PCR analysis of genes, miR-30a-5p and its precursors in xenograft tumors. **G** Detection of miR-30a-5p by RNA FISH in xenograft tumors. Scale bars: 200 and 100 μm. Values are means ± SE from *n* = 3 independent repetitions, *p ≤ 0.05, **p ≤ 0.01, and ***p ≤ 0.001 based on two-tailed Student’s *t*-test.

The in vivo experiments were performed utilizing a xenograft mouse model, which was established by subcutaneously injecting NCAPD3-stable overexpression and corresponding control PC-3 cells, respectively. We found that the size and weight of xenograft tumors of the NCAPD3-overexpression group were larger when compared to the control group (Fig. 7A–C; Supplementary Fig. 4A). Consistent with the in vitro results, we found that NCAPD3 overexpression led to a significant upregulation of STAT3, MYC, EZH2, and MALAT1 in tumors using qRT-PCR, Western blotting and IHC staining (Fig. 7D–F). However, miR-30a-5p was significantly decreased in the NCAPD3 overexpression group (Fig. 7F, G). PCLAF has been reported as a miR-30a-5p target gene [24], which was detected to be increased, corresponding to the reduction of miR-30a-5p in the NCAPD3-overexpression group (Fig. 7D, E). What’s more, pri-miR-30a, pre-miR-30a, and LINC00472 also had lower expressions in the NCAPD3-overexpression xenograft group compared to the control, which was consistent with the lower expressions of miR-30a-5p.(Fig. 7F). Lastly, NCAPD3-stable knockdown 22Rv1 cells and the control were separately applied to subcutaneous injection for constructing a mouse model. Interestingly, the tumors were only formed in the control group, and no signal was observed in the NCAPD3 knockdown group (Supplementary Fig. 4B–D). The results demonstrated that NCAPD3 promoted tumor growth in prostate cancer through down-regulating miR-30a-5p, which was associated with the regulation of STAT3, MYC, and EZH2.

## Discussion

Prostate cancer is one of the most common malignant tumors in men, ranked sixth in incidence (7.1%) and eighth in mortality (4.6%) in China during 2000–2016 [25]. Although AR-targeted therapies are initially effective, CRPC remains incurable [26], so it is imperative to identify the mechanisms underlying carcinogenesis and establish efficacious therapeutic targets. Due to a more profound understanding of gene regulatory mechanisms in recent years, non-coding RNA-related research has become increasingly prominent, and identifying the key points and axes in the ceRNA network has been regarded as a breakthrough point in discovering new candidate biomarkers and therapeutic targets for cancers. In this study, we identified that miR-30a-5p was experimentally downregulated by NCAPD3, following the bioinformatics analyses, and further demonstrated the low expression of miR-30a-5p in PCa cell lines and tissues.

miR-30a-5p is located in human chromosome 6q13 [27]. It belongs to a member of the miR‐30 family, including miR‐30a, miR‐30b, miR‐30c, miR‐30d, and miR‐30e with the same seed sequence, and each miRNA gene can produce two mature miRNAs (3p or 5p). miR-30a-5p has been found to be downregulated in several human cancers, including thyroid anaplastic carcinoma, endometrial cancer, and gallbladder cancer [28–30]. miR-30a-5p was involved in the regulation of tumor growth by controlling the expression of important oncogenes, e.g. miR-30a-5p reduced CD73 expression by targeting its 3’-UTR and inhibited lung cancer cell growth [31], and miR-30a-5p also directly interacted with YAP1 to modulate cell autophagy, invasion, and apoptosis in gastric cancer [32]. Notably, miR-30a was also reported to inhibit cell growth in PCa cells without expressing androgen receptors by targeting MYBL2, FOXD1, and SOX4 [33] and reduced tumorigenicity in vivo models of PCa [34]. This differed from our result that NCAPD3 inhibited miR-30a-5p expression, which was regulated by AR signaling, indicating antitumor miR-30a might function in both androgen-dependent and independent states associated with prostate cancer progression.

**Fig. 8:**
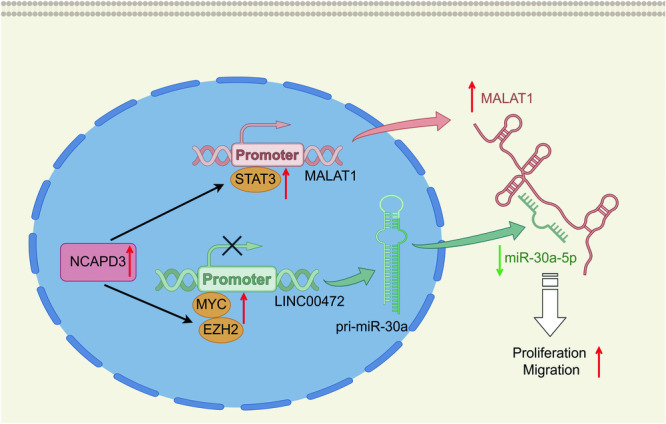
Schematic diagram of the mechanism of NCAPD3 regulated miR-30a-5p in PCa. NCAPD3 promoted lncRNA MALAT1 expression via upregulating STAT3, which binds the MALAT1 promoter region to activate transcription; however, MALAT1 silenced miR-30a-5p by acting as an endogenous miRNA sponge. Additionally, NCAPD3 also increased transcription factor MYC expression, and MYC directly decreased miR-30a-5p expression via binding its host gene LINC00472 promoter region to inhibit transcription initiation. The combination of these mechanisms caused miR-30a-5p not to exert tumor-suppressive effects, thereby accelerating tumor proliferation and migration. The graph was drawn by Figdraw.

We demonstrated that NCAPD3 had a dual impact to silence miR-30a-5p, leading to the acceleration of tumor development as shown in Fig. 8. On one hand, NCAPD3 upregulated STAT3 to bind the MALAT1 promoter region for inducing its expression, and MALAT1 functioned as a competing endogenous RNA to sponge miR-30a-5p. On the other hand, NCAPD3 increased the expression of transcription factor MYC, which directly inhibited miR-30a-5p transcription via binding its host gene LINC00472 promoter region, both of which promoted cell proliferation and migration and accelerated tumor growth in PCa. The MALAT1 gene is located in chromosome 11q13 and produces a well-conserved non-coding transcript that is over 8000 nucleotides in length [35]. MALAT1 was overexpressed in human prostate cancer and positively correlated with Gleason score, and cell proliferation and migration were inhibited in DU145 and PC3 cells when it was knocked down [36, 37]. MALAT1 was also highly expressed in other various cancers like non-small cell lung cancer [38] and melanoma [39], which is involved in a wide range of biological and cellular processes, including glycolysis and vascular growth, for promoting tumor progression [40]. The widely recognized mechanism of these effects was that MALAT1 can act as a ceRNA for miRNA like miR-23b-3p to attenuate the inhibitory effect of miRNA, leading to chemo-induced autophagy and chemoresistance in gastric cancer cells [41]. MALAT1 also triggered malignant melanoma growth and metastasis by sponging miR-22, consequently upregulating the miR-22 targets MMP14 and Snail [39]. Moreover, MALAT1 abolished miR-101-dependent negative regulation of the autophagic program in glioma cells by sponging miR-101, thereby prompting cell proliferation [42]. These revealed that MALAT1 contains multiple miRNA binding sites that contribute to sponge and regulate these miRNA expression and activity. According to target-directed miRNA degradation (TDMD) theory, extensive pairing between miRNA and targets promotes the dislocation of the 3’ end of miRNA from AGO2 binding, resulting in miRNA decay [43, 44]. It may also be the possible reason for MALAT1 decreasing miR-30a-5p expression, and the precise molecular mechanism remained to be further determined.

Apart from NCAPD3 regulation of STAT3, we also reported before that NCAPD3 upregulated other TFs like MYC and EZH2 to promote tumor progression [45]. Herein, we found that MYC was upregulated to inhibit miR-30a-5p after NCAPD3 was overexpressed, which was consistent with MYC inhibition of miR-30a in B and T cell malignancies, resulting from NOTCH1/2 induction [46]. MYC represses miR-34a and let-7 to accelerate the tumor by binding respective miRNA promoters as well [47]. Interestingly, we verified EZH2, similar to MYC, was upregulated to suppress miR-30a-5p expression by NCAPD3 overexpression, but did not find the binding sites between EZH2 and miR-30a-5p, implying EZH2 did not regulate miR-30a-5p level as a transcriptional factor. Actually, MYC can recruit EZH2 to miR-26a promoter and cooperatively repress miR-26a expression in lymphoma cells [48]. Likewise, EZH2 might be recruited by MYC for the co-inhibitory effect of miR-30a-5p, resulting from NCAPD3 overexpression.

The biogenesis of miRNAs is a sequential process: transcription of pri-miRNA, pri-miRNA processing to pre-miRNA, and pre-miRNA processing to mature miRNA [49]. Usually, miRNA genes are located throughout the genome, either within intronic sequences of protein-coding genes, within intronic/exonic regions of non-coding RNAs, or between independent transcription units (intergenic) [50]. So, many of them have their own promoters, some share promoters with host genes, and others are co-transcribed as a single primary miRNA transcript [51]. Since miR-30a is an intronic miRNA located in LINC00472, we therefore considered that LINC00472 should share the same promoter as miR-30a, which was also verified by the consistent trend of LINC00472, pri-miR-30a, pre-miR-30a, and miR-30a-5p expressions.

In conclusion, we found NCAPD3 inhibited miR-30a-5p expression to promote cell proliferation and migration and accelerate tumor growth through MALAT1 as a sponge and MYC transcriptional repression pathways. This finding provided a deeper insight into molecular mechanisms of cancer-promoting effects of NCAPD3 in prostate cancer.

## Materials and methods

### Clinical specimen

Prostate cancer samples were gathered from 20 individuals who had surgery at the Affiliated Hospital of Nanjing University of Chinese Medicine. Serum prostate-specific antigen (PSA), digital rectal examination, and histopathological examination of needle biopsy were used to detect and confirm all patients, with grading based on the Gleason score (Supplementary Table 1). Initially, individuals between the ages of 60 and 70 were diagnosed as patients, and it was confirmed that they had not undergone any radiotherapy or chemotherapy before the surgical procedure. This work was approved by the Ethics Committee of the Affiliated Hospital of Nanjing University after obtaining prior consent from every patient. Newly excised tissues were either stored at −80 °C or preserved in formalin and then embedded in paraffin.

### Cell culture, transfection

Four human prostate cancer cell lines (PC-3, DU 145, 22Rv1, and LNCaP) and an immortalized prostate stromal cell line (WPMY-1) were purchased from and checked by the National Collection of Authenticated Cell Cultures (Shanghai, China) and benign prostate hyperplasia cell line (BPH-1) was obtained from American Type Culture Collection. LNCaP cells were grown in Corning® CellBIND® dishes, and other cells were seeded in standard TC-treated cell culture dishes. To conduct experiments with androgen deprivation, CSS (Charcoal-stripped fetal bovine serum, GIBCO) rather than FBS was added in phenol red-free RPMI 1640 medium. NCAPD3 was stably overexpressed in PC-3 cells and knocked down in 22Rv1 cells, as previously reported [15]. Stable NCAPD3 overexpression PC-3 cells or stable NCAPD3 knockdown 22Rv1 cells and respective control cells were cultured in a medium containing 2 µg/mL puromycin and confirmed through RT-PCR and Western blotting each time before use.

Lipofectamine 2000 (Invitrogen, USA) was used for the transient transfections, following the guidelines provided by the manufacturer. GenePharma (Shanghai, China) provided the siRNA oligonucleotides for NCAPD3, STAT3, AR, MYC, EZH2, and miRNA mimics, which can be found in Supplementary Table 2.

### Antibodies and reagents

The primary antibodies for NCAPD3 and PCLAF were obtained from Santa Cruz Biotechnology (CA, USA). Other primary antibodies for STAT3, AR, MYC, EZH2, AGO2, Ki67, β-actin and the secondary antibodies, including HRP Goat Anti-Mouse IgG and HRP Goat Anti-Rabbit IgG, were obtained from ABclonal Technology (Hubei, China). MALAT1-IN-1 and Stattic were purchased from MedChemExpress (NJ, USA). Mibolerone (Mib) was purchased from Sigma Aldrich (MO, USA). Charcoal-stripped serum (CSS) was purchased from Biological Industries (Beit Haemek, Israel). Phenol red-free RPMI 1640 was purchased from Gibco (New York, USA).

### Cell viability assay

In CCK8 experiments, cells were distributed into 96-well plates at 3000 cells per well and incubated in an incubator for 24 h after dispensing. After applying the appropriate treatments for the specified duration, 10 μL of CCK8 (Beyotime, China) was introduced into every well. Then, incubation was continued for 2 h, and optical absorbance at 450 nm (OD_450_) was assessed using a microplate reader (Bio-Tek, USA).

To conduct the colony formation assay, a six-well plate was used to seed 400 cells per well with various treatments, followed by an incubation period of two weeks. Finally, the cells were washed three times using phosphate-buffered saline (PBS) and then fixed with 4% paraformaldehyde for a duration of 20 min. Following three washes with PBS, the cells were then treated with a 0.1% solution of crystal violet for 20 min. Scanning the plates allowed for the determination of colony sizes and the measurement of quantities. A microscope was used to observe and photograph colonies.

### Cell motility assay

To perform the wound healing assay, cells subjected to various treatments were placed in six-well dishes and grown until they reached 80% confluence. Afterward, the layer of cells was scraped using a sterile micropipette tip with a volume of 10 μL and rinsed two times before being kept in serum-free media. Images of the identical wound region were captured with an optical microscope (Olympus, Japan) at 0 and 24 h. The formula used to calculate the relative migration distance is the percentage of wound closure (%) = 100(*A*−*B*)/*A*. Here, *A* denotes the initial width of cell scratches prior to incubation, while *B* represents the width after incubation.

To perform the migration test, the cells were placed in a culture medium containing 0.2% FBS and then added to the Transwell chambers (8 μm, Corning, USA) at a density of 5 × 10^4^ cells per well. After 16 h, the chambers underwent three rounds of washing, and the cells on the opposite side were subsequently scraped away. Next, the cells were treated with 4% paraformaldehyde, followed by staining with Giemsa solution for a duration of 10 min. After air drying, the cells were photographed, and the count was performed.

### Western blotting (WB)

To perform the western blotting, we utilized RIPA lysis buffer enriched with protease inhibitors (Beyotime, China) and phenylmethylsulfonyl fluoride (Sigma, USA) to extract total proteins. The protein quantification was carried out using a BCA Protein Assay Kit (Beyotime, China), and equal amounts of the protein samples were loaded onto a 10% SDS–PAGE gel and subsequently transferred onto PVDF membranes (Millipore, USA). A solution of 5% non-fat powdered milk (Sangon, China) in PBS was applied at room temperature for 1.5 h to block the membranes. Subsequently, the membranes were incubated overnight at 4 °C with the appropriate primary antibody. After three washes with PBST (PBS containing 0.1% Tween-20), the membranes were incubated with secondary antibodies for 1 h at room temperature. Following another round of washing with PBST, the signals of the target proteins were detected using an enhanced chemiluminescence detection kit (Tanon, China). Quantitative analysis for Western blotting outcomes was carried out using ImageJ software from the National Institutes of Health in the United States.

### Quantitative real-time PCR (qRT-PCR)

TRIzol reagent (Life Technologies, USA) was utilized to extract the total RNA following the manufacturer’s instructions. PrimeScript RT-PCR Kit (TaKaRa, Japan) was employed to synthesize cDNA, and qRT-PCR was conducted using qPCR SYBR Green Master Mix (Vazyme, China). The 2^−∆∆Ct^ method was used to analyze mRNA levels after normalizing with β-actin. Supplementary Table 4 contains the sequences of the primers used in the experiments.

To analyze small RNAs, the cells or tissues were lysed using Trizol Reagent and then stored at −80 °C overnight to ensure successful RNA extraction of excellent quality. The cDNA synthesis and qPCR analysis of miRNA were carried out according to the procedure provided by the TransScript® Green miRNA Two-Step RT-PCR SuperMix kit (TransGen Biotech, China). The reactions were replicated thrice. The 2^−ΔΔCT^ method was utilized to compute the relative expression levels of every miRNA.

### Immunohistochemistry (IHC) assay

Sections of tissue fixed with paraformaldehyde and embedded in paraffin were cut to a thickness of 4 μm. The samples were subjected to a drying process at a temperature of 62 °C for 2 h. They were then treated with xylene to remove wax and then gradually rehydrated using alcohols of varying concentrations. The activity of internal peroxidases was suppressed with 3% hydrogen peroxide for 20 min at room temperature. The slides were boiled in a microwave oven for 15 min using a 0.01 mol/L sodium citrate buffer (pH 6.0) to retrieve the antigen. To prevent the binding of non-specific antigens, 10% goat serum in PBS was used for incubation for 30 min. After being exposed to primary antibodies for ~12 h at a temperature of 4 °C, the slides underwent rinsing with PBS 3 times, each lasting 5 min, at room temperature. Subsequently, the detection of antibody binding was carried out using a SABC-POD (F) rabbit IgG kit (Boster, China). Microscopic examination was conducted on stained sections and documented using a Nikon microscope from Japan. The ImageJ program (NIH, USA) was utilized to measure the relative intensities of IHC staining.

### Dual-luciferase reporter assay

Recombinant vectors of the MALAT1 promoter and the mutations of the miR-30a-5p binding site in the MALAT1 promoter (pmirGLO-MALAT1-promoter-wt/mut) were obtained from Tsingke Biotechnology (Beijing, China). We inserted the promoter sequence of pri-miR-30a into the pGL3/luciferase basic vector to construct pGL3/luciferase-pri-miR-30a (pGL3-miR-30a). For dual-luciferase reporter assay, the cells were seeded into 96-well plates and co-transfected with dual-luciferase reporter vectors and miR-30a-5p mimics or miR-NC by using Lipofectamine™ 2000. Following a 48-h incubation period, the double luciferase reporter gene detection kit (Beyotime, China) was utilized to assess the firefly and renilla luciferase activities. The mean ± SE of firefly/renilla luciferase activity in relative light units (RLU) was used to normalize the results.

### RNA-seq and miRNA-seq analysis

Three pairs of prostate cancer tissues and adjacent normal tissues were stored in liquid nitrogen, and three replicate dishes of each cell type (PC-3-Lv-Control and PC-3-Lv-NCAPD3, 22Rv1-Lv-shControl and 22Rv1-Lv-shNCAPD3) were lysed in Trizol Reagent and stored at −80 °C. Total RNA isolation, RNA-seq, and miRNA‐seq library construction, and sequencing were performed at Majorbio (Shanghai, China). Sequencing reads were quality-filtered with Fastx-Toolkit (www.hannonlab.cshl.edu); clean reads were obtained by removing the adapter sequences and filtering low-quality sequences at the end of the reads. Filtered fastq files were aligned to the human reference genome (GRCh38.p13), read mapping was performed with Hisat2 (http://daehwankimlab.github.io/hisat2/) for RNA-Seq, and known miRNAs were verified using miRDeep2 (http://www.mdc-berlin.de/rajewsky/miRDeep). The differentially expressed genes and miRNAs were analyzed by R package DESeq2 (https://bioconductor.org/packages/release/bioc/html/DESeq2.html).

### RNA Immunoprecipitation (RIP)

The cellular lysates were sonicated. Immunoprecipitation was performed 12 h at 4 °C with 5 μg Argonaute 2 (AGO2, ABclonal, China) monoclonal antibody or the control isotype IgG (ABclonal, China) and then with protein A/G-Sepharose. Removing the supernatant after centrifugation, the magnetic beads were washed for 5 min, and the supernatant was discarded again. The magnetic beads were eluted with nuclease-free water, and eluates were pooled and used as a template for qRT-PCR.

### Fluorescence in situ hybridization (FISH)

The fluorescence in situ hybridization assay was performed using the Fluorescence in Situ Hybridization Kit (GenePharma, China). miR-30a-5p was captured with Cy3-labeled probes, and sequences of probes were shown in Supplementary Table 2. For paraformaldehyde-fixed paraffin-embedded sections, the sections were heated at 60 °C for 30 min, and fresh xylene was applied to eliminate the paraffin from the tissue. Next, the sections were rehydrated through 5 min incubations in ethanol with decreasing concentrations. Following that, they were exposed to proteinase K solution for 20 min at a temperature of 37 °C and denaturing solution for 8 min at 78 °C. Sections were then dehydrated by washing in a series of ethanol, and the denatured probes in fresh hybridization solution were added, and the mixture was incubated at 37 °C overnight. For cell-climbing slices, plates were rinsed three times in PBS, fixed in paraformaldehyde for 15 min, permeabilized in 0.1% Triton X‐100 for 15 min, washed three times in PBS, and incubated with blocking solution for 30 min. As above, the denatured probes were then added, and the mixture was incubated at 37 °C overnight. Nuclei were marked by staining with DAPI. Images were acquired using a fluorescence microscope (Olympus, Japan).

### In vivo experimental assay of transplanted tumor in nude mice

NCAPD3-stable overexpression PC-3 cells (2 million) or NCAPD3-stable knockdown 22Rv1 (5 million) cells and their corresponding control cells were suspended in 100 μL of PBS and subcutaneously injected into the left axillae of male BALB/c nude mice (GemPharma, China) aged 5 weeks. The mice were subsequently maintained in a pathogen-free environment. Each group had approximately five or six mice. The tumor volume was measured using a caliper every 2 days, starting 7 days after the subcutaneous inoculation. The equation *ν* = ½*ab*^2^ (where *a* represents the longitudinal diameter and *b* represents the latitudinal diameter) was utilized to compute the volumes of the tumors. Following a period of 6 weeks, the mice were euthanized and separated, and their xenograft tumors were measured in terms of weight. These tumors were then either fixed with paraformaldehyde or frozen for future use in the experiment. The Experimental Animal Ethics Committee of Nanjing Normal University approved all animal studies and ensured compliance with the National Guidelines for Animal Usage in Research (China).

### Statistical analysis

Mean ± SEM is used to present the results. The significance among groups was assessed using the SPSS statistical package (SPSS, IL, USA) and GraphPad Prism 8 software (CA, USA). This was done by employing the Pearson chi-squared test, two-tailed Student’s *t*-test, Kaplan–Meier plot, or ANOVA analysis, as deemed suitable. Statistical significance was determined at a value of *P* < 0.05, with single, double, and triple asterisks representing *P < 0.05, **P < 0.01 and ***P < 0.001, respectively.

### Supplementary information

Supplementary materials

Original Data File

## Data Availability

All data generated or analyzed during this work are included in this published article and its supplementary information files.
