# Supplementary material for: NCAPD3 exerts tumor-promoting effects in prostatic cancer via dual impact on miR-30a-5p by STAT3-MALAT1 and MYC
Source: Cell Death Discov. 2024 Apr 1;10:159. doi: 10.1038/s41420-024-01930-7 (PMC10985108; doi:10.1038/s41420-024-01930-7)
Supplement: Supplementary file 1 — Supplementary materials [file 41420_2024_1930_MOESM1_ESM.pdf]

## Supplementary Figure 1

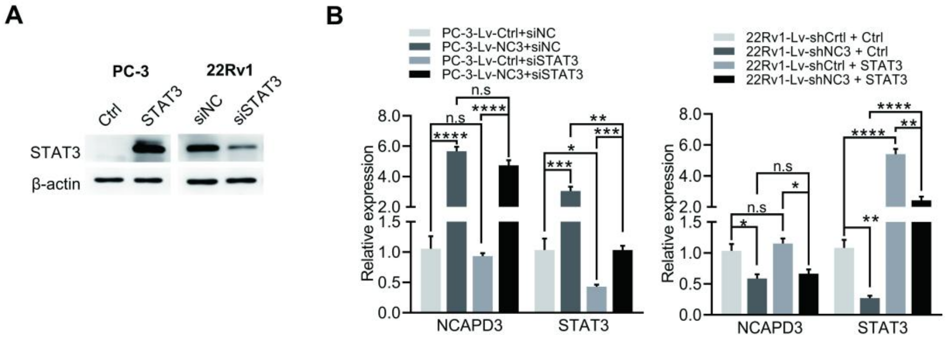


**A** The efficiency of STAT3 silencing in Fig.1H was demonstrated by Western blotting. **B** The knockdown and overexpression efficiency of NCAPD3 and STAT3 (Fig.1I ) were quantified by qPCR assay.

## Supplementary Figure 2

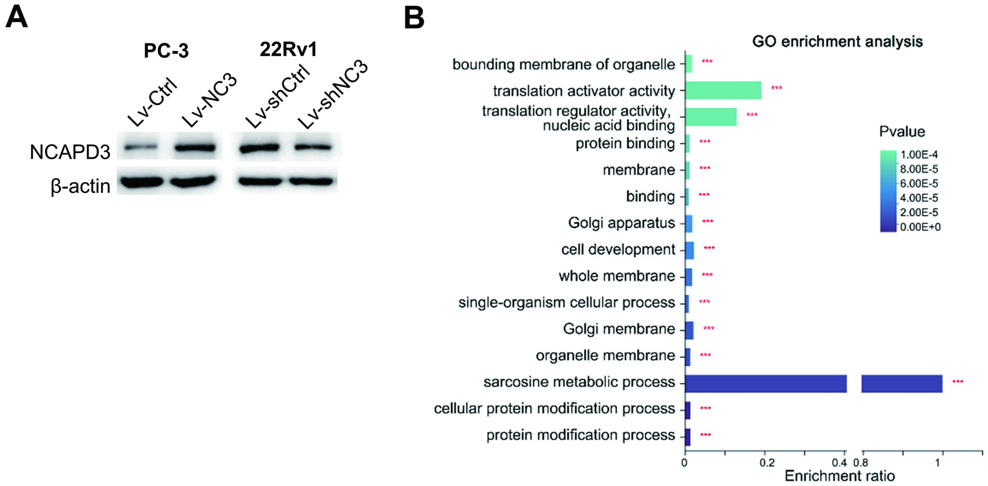


**A** The expression of NCAPD3 in overexpression-stable PCa cell lines and silent stable PCa cell lines of NCAPD3 was demonstrated before miRNA sequencing by Western blotting. **B** Bar charts of GO enrichment analysis of target genes of miR-30a.

## Supplementary Figure 3

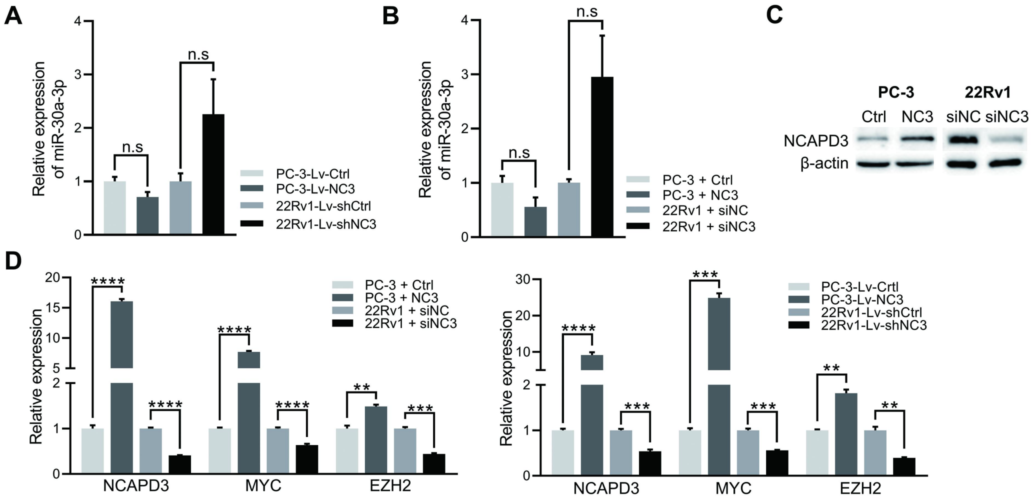


**A-B** qRT-PCR analysis of miR-30a-3p expression in stable NCAPD3 overexpression or knockdown cells (A) and transiently transfected cells with NCAPD3 plasmid or siRNA (B). **C** NCAPD3 and STAT3 knockdown and overexpression efficiency (Fig.3B) were quantified by Western blotting. **D** Wester blotting quantification of Fig.5F was performed using ImageJ.

## Supplementary Figure 4

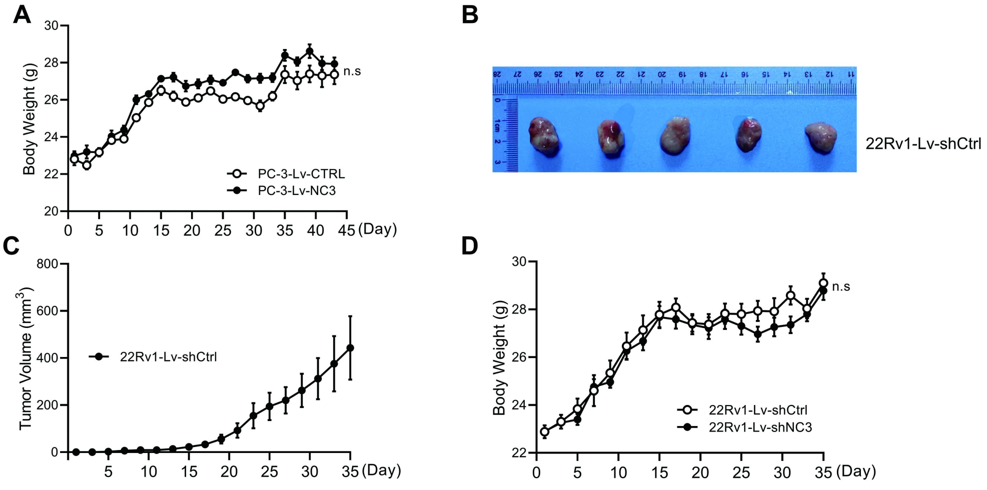


**A** Plot of weight changes of the xenografted nude mouse in stable NCAPD3 overexpression and control group. **B** Tumor volume changed in 22Rv1-Lv-shControl xenograft tumors nude mice. **C** Average tumor volume of each mouse group was measured and presented. **D** Plot of weight changes of the xenografted nude mouse in the 22Rv1-Lv-shControl group.

**Supplementary Table 1 Clinical information for the patient.**

| Number | Age (Years-old) | Gleason score | Median PSA (ng/mL) |
|---|---|---|---|
| 5 | 60 | Aggressive (7) | 68.9 |
| 15 | 68.8 | Very aggressive (8−10) | 74.74 |

**Supplementary Table 2 The sequences of oligonucleotides.**

| **siRNA** | **Sequences (5’-3’)** |
|---|---|
| negative control siRNA | UUCUCCGAACGUGUCACGUTT |
| NCAPD3 siRNA | AGGAAUUCAAGUUAACAGAGGCUUG |
| STAT3 siRNA | GCAACAGAUUGCCUGCAUUTT |
| AR siRNA | AAGAAGGCCAGUUGUAUGGAC |
| MYC siRNA | CGAUGUUGUUUCUGUGGAATT |
| EZH2 siRNA | GCUCCUCUAACCAUGUUUATT |
| miR-30a-5p mimics | UGUAAACAUCCUCGACUGGAAG |
| miR-NC | ACAUAGGGCCCAUGCUAACUGC |
| miR-30a-5p FISH Probe | CTTCCAGTCGCGGATGTTTACA-Biotin |

**Supplementary Table 3 An informative list of antibodies and inhibitors.**

| **Antibodies** | **Catalog No.** | **Manufacturer** |
|---|---|---|
| β-actin | AC006 | ABclonal |
| NCAPD3 | sc-101016 | Santa Cruz |
| STAT3 | A1192 | ABclonal |
| AR | A19611 | ABclonal |
| MYC | A1309 | ABclonal |
| EZH2 | A19577 | ABclonal |
| AGO2 | A19709 | ABclonal |
| PCLAF | sc-390515 | Santa Cruz |
| Ki67 | A11390 | ABclonal |
| IgG | A19709 | ABclonal |
| HRP Goat Anti-Rabbit IgG (H+L) | AS014 | ABclonal |
| HRP Goat Anti-Mouse IgG (H+L) | AS003 | ABclonal |
| MALTA1-IN-1 | HY-115579 | MCE |
| Stattic | HY-13818 | MCE |

**Supplementary Table 4 The primers used for qRT-PCR assay.**

| **Primer** | **Sequences (5’- 3’)** |
|---|---|
| β-actin | F-GAGCTACGAGCTGCCTGACG<br>R-CCTAGAAGCATTTGCGGTGG |
| NCAPD3 | F-TGGAGCAAGAGTCGAATGGCG<br>R-GGGGCGGTTTATCAGGCAGTG |
| STAT3 | F-GCTGCCCCATACCTGAAGA<br>R-AAACTGCCCTCCTGCTGAG |
| AR | F-ATGGTGAGCAGAGTGCCCTATC<br>R-ATGGTCCCTGGCAGTCTCCAAA |
| MYC | F-CCTGGTGCTCCATGAGGAGAC<br>R-CAGACTCTGACCTTTTGCCAGG |
| EZH2 | F-AAAGGAGTTTGCTGCTGCTC<br>R-TGTTATTGGGAAGCCGTCCT |
| MALAT1-3’ | F-AAAGCAAGGTCTCCCCACAAG<br>R-GGTCTGTGCTAGATCAAAAGGCA |
| MALAT1-5’ | F-GAATTGCGTCATTTAAAGCCTAGTT<br>R-GTTTCATCCTACCACTCCCAATTAAT |
| miR-30a-5p | F-CGCCGCTGTAAACATCCTCGAC<br>R-ATCCAGTGCAGGGTCCGAGG |
| miR-30a-3p | F-CGCCGCGCTGCAAACATCCGACT<br>R-ATCCAGTGCAGGGTCCGAGG |
| U6 | F-CTCGCTTCGGCAGCACA<br>R-AACGCTTCACGAATTTGCGT |
| pri-miR30a | F-GTAACCTGGCCACTTGCCTA<br>R-TCATCACCGAGCTTCTAGTTTCT |
| pre-miR30a | F-GCGACTGTAAACATCCTCGAC<br>R-AGCTGCAAACATCCGACTGA |
| LINC00472 | F-GGAGATACGGTCAGAGAGGC<br>R-GGTGCTTCATAGGGGTCTGT |

**Supplementary Table 5 The primers used for ChIP assay.**

| Gene | Sequences (5’- 3’) |
|---|---|
| MALAT1 promoter | F-CATGCCATTCCCCAGAACAGGC<br>R-CCGCGCAGGGATACGCGA |
