# Supplementary material for: NCAPD3 exerts tumor-promoting effects in prostatic cancer via dual impact on miR-30a-5p by STAT3-MALAT1 and MYC
Source: Cell Death Discov. 2024 Apr 1;10:159. doi: 10.1038/s41420-024-01930-7 (PMC10985108; doi:10.1038/s41420-024-01930-7)
Supplement: Supplementary file 2 — Original Data File [file 41420_2024_1930_MOESM2_ESM.docx]

F1D


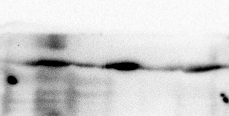

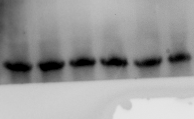
NCAPD3 β-actin

F1F

PC3 22Rv1


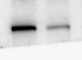

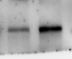
NCAPD3


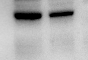

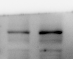
STAT3


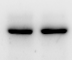

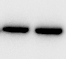
β-actin

F3B


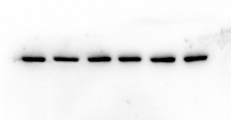

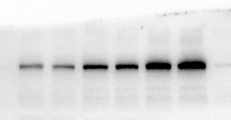
NCAPD3 β-actin

F3I


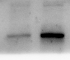

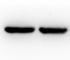

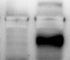
AR NCAPD3 β-actin

F3K


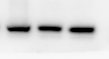

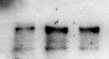

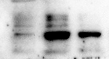
AR NCAPD3 β-actin

F5B


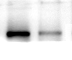
PC3 22Rv1


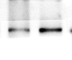
MYC


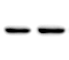

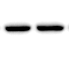
β-actin


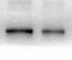

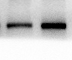


EZH2


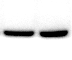


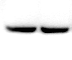
β-actin

F5F

PC3 22Rv1


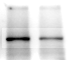

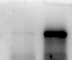
NCAPD3


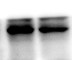

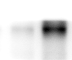


MYC


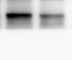

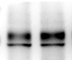
EZH2


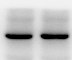

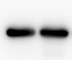
β-actin

F5G

PC3 22Rv1


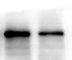

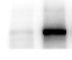
NCAPD3


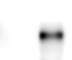


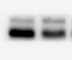
MYC


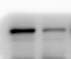


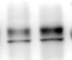
EZH2


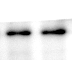


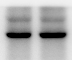
β-actin


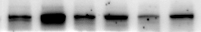
F7C

NCAPD3


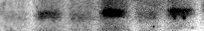
PCLAF


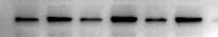
STAT3


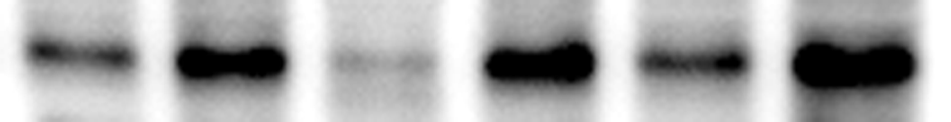
MYC


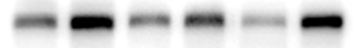
EZH2


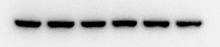


β-actin

SF1A


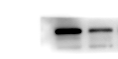

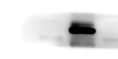


STAT3


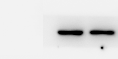

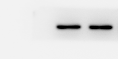


β-actin

SF2A


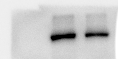

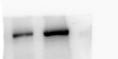


NCAPD3


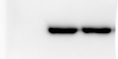

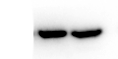


β-actin

SF3C


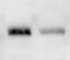

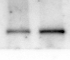
NCAPD3


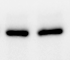

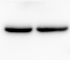
β-actin
